# Research in burns – Present and future

**DOI:** 10.4103/0970-0358.70717

**Published:** 2010-09

**Authors:** Andrew Burd

**Affiliations:** Division of Plastic, Reconstructive and Aesthetic Surgery, Department of Surgery, The Chinese University of Hong Kong, Prince of Wales Hospital, Hong Kong

**Keywords:** Immunology, face transplant, global networks, burns care

## Abstract

There have been tremendous advances in burns care over the past 50 years. Much of this, but not all, can be attributed to basic science and clinically related research. Out of the best centres in the world, centres that are fully funded and richly resourced, best practice guidelines result in impressive outcomes not only in terms of survival but also in terms of a quality of survival. Indeed the remaining clinical challenges in these centres are the elderly, the inhalational burns, and the very extensive burns. There are however other challenges when looking at burns care in a global context and in particular is the provision of even minimal standards of acceptable care for burns patients in many parts of the world. Whilst the justification for research funding in the wealthy countries becomes increasingly esoteric, for example looking at the immunology of face transplantation, the global health challenges of burns care still remain. Perhaps, the greatest research challenge in burns care in the 21st century lies not in furthering our understanding of the phenomenon we observe but the global application of the knowledge we already possess.

## PAST AND PRESENT

Fifty years ago, the first International Congress on Research in Burns was held at the National Navel Medical Centre in Bethesda, Maryland, USA. A compilation of 64 papers was edited by Artz and published as the proceedings.[1] The topics covered included incidence, pathophysiology, and prognosis, fluid and metabolic changes, infection as well as general and local treatments. The last 50 years have seen tremendous advances in the management of the acute burn, and in the last decade these improvements have been documented in a series of annual reviews entitled “What’s New in Burns and Metabolism” published by the Journal of the American College of Surgeons. Table 1 is extracted from a paper published in *Surgical Practice* that reviewed the state of burns care in Hong Kong in 2005.[2] This paper focussed on the following areas:

**Table 1:** The world underwent a dramatic change during the early years of the first decade of the 21^st^ century. Disaster planning is an emerging issue

*Reference no.*	*1*	*2*	*3*	*4*	*5*	*6*
Author	Hasselgren PO	Luterman A	Cioffi WG	Heimbach D	Saffle JR	Sheridan RL, Tompkins RG
Year	1999	2000	2001	2002	2003	2004
Paper	Burns and metabolism	Burns and metabolism	What’s new in burns and metabolism	What’s new in general surgery: Burns and metabolism	What’s new in general surgery: Burns and metabolism	What’s new in burns and metabolism
Topics covered				√	√	√
Organization and delivery of care				√	√	√
Verification				√	√	√
Burns centers				√	√	√
Disaster planning				√	√	√
Metabolic response to injury	√					
Inhalational injury and co- poisons		√		√	√	√
Burn resuscitation		√	√		√	√
Pain management				√		√
Nutritional support	√	√	√	√	√	√
Glutamine	√	-	-	-	√	-
growth hormone	√	-	√	-	-	-
IGF-1	√	√	√	-	√	-
oxandrolone	-	√	√	-	√	-
Burn wound management		√		√	√	√
Skin substitutes		√		√	√	√
Healing and scarring		√	√	√	√	√
Rehabilitation		√		√	√	√
Reconstruction					√	√
Non-burn wounds					√	√
No of references	49	88	77	86	166	366

- Prevention of burns
- Organization and delivery of burns care
- Decompression instead of escharotomy as a conceptual process
- Fluid resuscitation
- The burn wound excision and closure
- The management of chemical assault burns
- Infection control and silver ions
- Microsurgical and advanced reconstructive techniques in burns care
- Tissue engineering and regeneration
- Finally, the Asia Pacific Burns Association

Meanwhile in the USA, the American College of Surgeons were running out of things to contribute to an annual feature and so in 2005, John Burke authored a paper looking at the “Evolution of burn care over the 20^th^ century” and focussed on the elimination of burn shock due to appropriate resuscitation and the early excision of the burn wound with immediate physiological wound closure as being the major contribution to improved outcome.[3] Indeed Burke stated that “At the close of the 20^th^ century, overall mortality had decreased to very low levels and considerable attention was given and improvement made, in the patient’s long-term cosmetic and functional quality of life. Nevertheless, optimal burn therapy has not been reached.” Burke suggested that the challenges for the 21^st^ century (this century) were going to focus on the elderly, inhalational burn and the very extensive injury.

Burke did not get it all his own way and a letter from a European surgeon raised the issue of money and resource allocation but also the use of cerium nitrate as an alternative to early excision.[4] Allgöwer presented the major challenges for the 21^st^ century as being improved care in the third world countries, and mass casualty management as in terrorist strikes.

Two articles in the Journal of Burn Care and Research added further perspectives; Holmes underlined the challenge of sustaining effective burns care in the United States due to multiple factors including shortages of trained staff, reduction in patient numbers and uneven distribution of burn care facilities.[5] All these are very real practical issues. On the other end of the spectrum was an interesting paper from Illinois looking at the potential implications of genomics in clinical practice.[6]

In the same year of publication of these two articles, in 2008, Steven Wolf, the editor of the Burns Journal, took up the annual review of the Burns world and began with “The year in burns 2007”.[7] Wolf indicated that approximately 1000 original articles related to burns research were published in English language scientific journals in 2007. These covered the main areas that are as follows:

- Burn epidemiology
- Wound characterization and treatment
- Critical care
- Inhalation injury
- Infection and inflammation
- Metabolism and nutrition
- Psychological considerations
- Pain
- Rehabilitation
- Reconstruction

**Figure 1:**
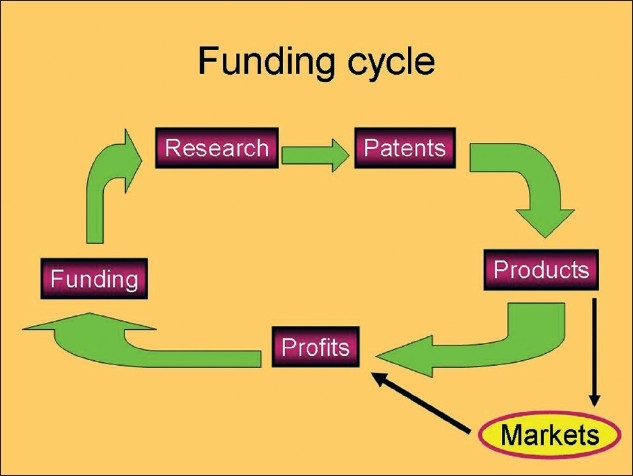
The funding cycle in the developed world where much of the drive has been from the commercial world

The exercise was repeated the following year and in 2008; approximately 1200 burns-related research articles were published in the English language.[8] We await with interest the next review in 2010; however, there are some important points to make – in the past, a considerable amount of burn research had a funding support from commercial sources who were driven by the potential financial rewards [Figure 1]. However, with the decreasing number of major burns in North America and Europe, this funding has been reducing. This may not necessarily be a bad thing as the reality is that in the best centres in the world; survival and quality of outcome are extremely good. The notion of 100% survival is now accepted as an unrealistic goal. The point being does “Burns” need more research? In global terms, if the whole field of basic science research in burns stopped tomorrow, would it have a catastrophic effect on the development of burns care worldwide?. Probably not. What would have an impact would be the wider, indeed global, application of what is already known. Whilst not diminishing the impact and importance of the work of many current investigators a review, for example, of the Burns journal for 2009 does not deliver any “Red Alert” new findings. Think for example about the letter to the Editor of the New England Journal of Medicine about the use of propranolol in infantile haemangioma[9] A serendipitous finding which is changing the management of haemangioma worldwide. What is obvious is that the exploration of the fundamental biology in burnsrelated pathophysiology continues whilst the clinical application is being more carefully assessed by outcome studies. We still do not understand why hypertrophic scars develop[10,11] but then does it really matter? We still have not found the perfect resuscitation formula[12,14] but does it really matter? We can look at some aspects of burns care that do matter and certainly outcomes are very important.[15] The management of pain is of critical significance in clinical burns care.[16] Return to work, another very practical aspect of burns research.[17]

## THE FUTURE

**Figure 2:**
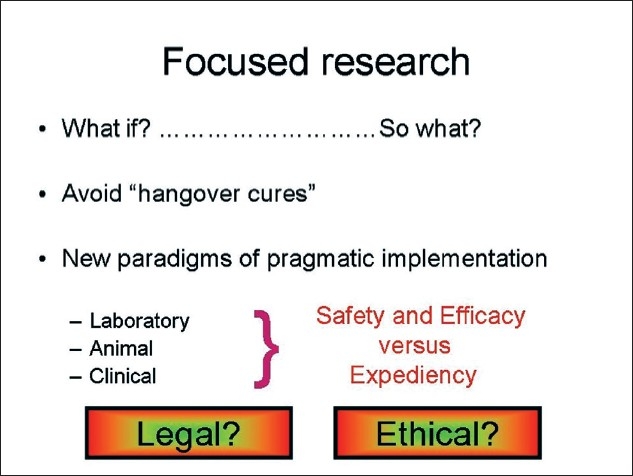
It is important to avoid research just for the sake of research. The global need in burns care is for immediate implementation of what is already known. How that might be achieved is more in the reality of policy and public health research

**Figure 3:**
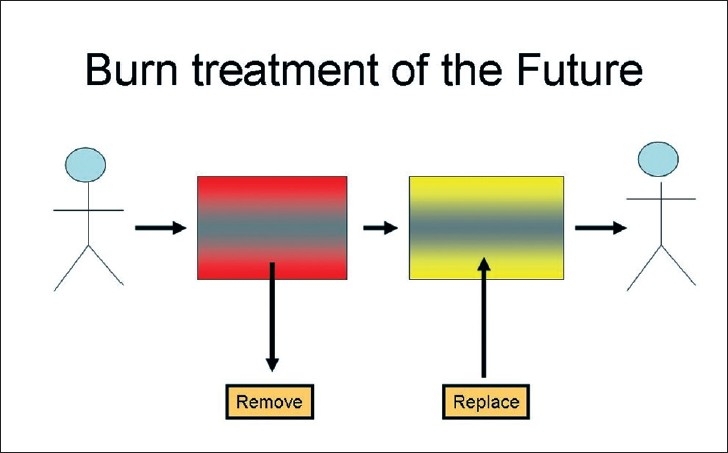
Burns: The challenge, remove and replace

But what are the challenges for the future in burns research? In Figure 2, I encapsulate the concept of focussed and relevant research. The methodology must be legal and ethical and the focus directed towards achieving what is safe and effective. Safe and effective in achieving what? Figure 3 illustrates the challenge of cutaneous burns in its most basic concept. We have a human being who sustains a burn injury; the burn tissue needs to be removed and replaced in such a way that the integrity, the form and function of the tissues are restored.

It is these aspects of burn care that are going to provide the most fruitful areas of research in the foreseeable future. Burke has made tremendous contributions in the field of tissue engineering. Transplantation has taken on a new dimension with the reality of facial transplantation being demonstrated around the world. But what is the major concern of burns and plastic surgeons towards such procedures? Not the procedure itself but the associated risks and complications of immunosuppression.[18] So researchers in the field of burns care are looking again at immunological aspects of repair and reconstruction, and the role of stem cell therapy will be of ever increasing interest not only from the perspective of basic science but also clinical research.[19,20]

As in many other aspects of health and disease, we have accumulated a considerable amount of knowledge and understanding about the biology of our challenge and interventions. That is the nature of burns and their treatment. So let me end on a suitably controversial note. Why not stop all basic science and clinical burns-related research in the Western world and redirect all the money, time, and effort into applying what we do know; to burns patients throughout the world. In order to achieve this, I propose a search committee that is convened to compile a list of criteria upon which Burns centres can be assessed and then, the guidelines and protocols of the top ten centres that could be used as templates for developing a global network of accredited burns centres. A dream or a mission? I am not sure, but certainly something worthy of research in terms of practical implementation, and certainly something to propose to the World Health Organization.
